# Supplementary material for: Individual experience as a key to success for the cuckoo catfish brood parasitism
Source: Nat Commun. 2022 Mar 31;13:1723. doi: 10.1038/s41467-022-29417-y (PMC8971504; doi:10.1038/s41467-022-29417-y)
Supplement: Supplementary file 1 — Supplementary Information [file 41467_2022_29417_MOESM1_ESM.pdf]

Supplementary Information for

Individual experience as a key to success for the cuckoo catfish brood parasitism

Holger Zimmermann, Radim Blažek, Matej Polačik & Martin Reichard

Supplementary Tables 1-11

Supplementary Figures 1-3

Supplementary Table 1: No effect of *A. burtoni* exposure to cuckoo catfish parasitism on catfish parasitism success. Table shows the results for the fixed effects of a GLMM (binomial error distribution) with the proportion of cuckoo catfish eggs found in cichlid clutches (modelled as catfish eggs vs. cichlid eggs) as response variable and time progress of the experiment (in days from 1 to 151) and female cichlid body length (mm) as predictor variables. 'Tank ID' was included as a random intercept to account for non-independence of data points in the same experimental tank. We also included an individual observation-level random intercept to account for overdispersion [63]. Data represents egg counts from 32 spawnings within two tanks over the course of 151 days. *A. burtoni* individuals showed no signs of avoidance to cuckoo catfish parasitism over the entire experimental period.

	Estimate	*Std. Error*	*z*	*P*
(Intercept)	-11.68	10.91	-1.07	0.285
Time progress of experiment	-0.02	0.03	-0.68	0.497
Cichlid female body size	0.08	0.16	0.54	0.592

Supplementary Table 2: Effects of cuckoo catfish experience and time progress on the likelihood of successfully parasitising a host female. Female total length was included as additional factor as it may affect catfish tendency to intrude on a specific cichlid spawning to attempt parasitism. Significant interaction terms indicate a decreasing difference in the probability of parasitism between naïve cuckoo catfish and their more experienced congeners over time. The upper part of the table presents the GLMM results with catfish experience level 'naïve' as model reference [set with R function 'relevel']. The lower part of the table shows the results of the same GLMM with model reference set to 'experienced' cuckoo catfish using the same R function. Significant p-values ($p < 0.05$) are in bold.

	Estimate	*Std. Error*	*z*	*P*
**Naïve catfish**				
(Intercept)	-5.43	1.85	-2.94	**0.0033**
Time progress of experiment	0.03	0.01	3.04	**0.0023**
Catfish experience – *experienced*	3.61	0.84	4.31	**< 0.0001**
Catfish experience – *highly-experienced*	3.13	0.87	3.60	**0.0003**
Cichlid female body size	0.02	0.02	0.95	0.3410
Time progress of experiment : *experienced* catfish	-0.04	0.01	-3.78	**0.0002**
Time progress of experiment : *highly-experienced* catfish	-0.04	0.01	-3.45	**0.0006**
**Experienced catfish**				
(Intercept)	-1.82	1.70	-1.07	0.2862
Time progress of experiment	-0.01	0.01	-2.15	**0.0320**
Catfish experience – *highly-experienced*	-0.49	0.65	-0.75	0.4518
Catfish experience – *naïve*	-3.61	0.84	-4.31	**< 0.0001**
Cichlid female body size	0.02	0.02	0.95	0.3410
Time progress of experiment : *highly-experienced* catfish	0.001	0.01	0.12	0.9070
Time progress of experiment : *naïve* catfish	0.04	0.01	3.78	**0.0002**

Supplementary Table 3: Effects of cuckoo catfish experience and time progress of the experiment on the mean number of cuckoo catfish eggs found in clutches of host females (i.e., total abundance). Female total length was added as additional factor that may affect catfish tendency to intrude on a specific cichlid spawning to attempt parasitism. Significant interaction terms indicate a decrease in the difference in the catfish egg numbers found in cichlid clutches between naïve cuckoo catfish and more experienced catfish as the experiment progressed. The upper part of the table shows the GLMM results with catfish experience level 'naïve' as model reference [set with R function 'relevel']. The lower part of the table shows the results of the same GLMM with model reference set to 'experienced' cuckoo catfish using the same R function. Significant p-values ($p < 0.05$) are in bold.

	Estimate	*Std. Error*	*z*	*P*
**Naïve catfish**				
(Intercept)	-2.95	1.55	-1.90	0.0571
Time progress of experiment	0.02	0.01	3.06	**0.0022**
Catfish experience – *experienced*	3.09	0.74	4.17	**< 0.0001**
Catfish experience – *highly-experienced*	2.80	0.77	3.63	**0.0003**
Cichlid female body size	0.01	0.01	0.49	0.6277
Time progress of experiment : *experienced* catfish	-0.03	0.01	-3.67	**0.0002**
Time progress of experiment : *highly-experienced* catfish	-0.03	0.01	-3.50	**0.0005**
**Experienced catfish**				
(Intercept)	0.15	1.42	0.11	0.9167
Time progress of experiment	-0.01	0.005	-1.94	0.0525
Catfish experience – *highly-experienced*	-0.29	0.53	-0.55	0.5835
Catfish experience – *naïve*	-3.09	0.74	-4.17	**< 0.0001**
Cichlid female body size	0.01	0.01	0.49	0.6278
Time progress of experiment : *highly-experienced* catfish	-0.001	0.01	-0.13	0.9004
Time progress of experiment : *naïve* catfish	0.03	0.01	3.67	**0.0002**

Supplementary Table 4: Effects of cuckoo catfish experience and time progress in the experiment on the mean clutch size of cuckoo catfish eggs in the set of parasitised clutches. Female total length was included as an additional factor that may affect catfish clutch size as the size of the host female defines the number of her eggs (see Supplementary Table 5) and therefore may increase the likelihood that cuckoo catfish deposit more eggs in the host clutch. Indeed, host female size did affect catfish clutch size significantly, but results indicate that host acceptance of catfish eggs was highest in small host females. One possible explanation could be that larger cichlid females were more vigorous in their defence and did not offer repeated chances for parasitism to catfish during repeated bouts of spawning. The upper part of the table shows the GLMM results with catfish experience level 'naïve' as model reference (set with R function 'relevel'). The lower part of the table shows the results of the same GLMM with model reference set to 'experienced' cuckoo catfish using the same R function. Significant p-values ($p < 0.05$) are in bold.

	Estimate	*Std. Error*	*z*	*P*
**Naïve catfish**				
(Intercept)	-3.86	1.20	3.21	**0.0014**
Time progress of experiment	-0.001	0.01	-0.08	0.9365
Catfish experience – *experienced*	-0.03	0.64	-0.04	0.9672
Catfish experience – *highly experienced*	-0.01	0.64	-0.01	0.9899
Cichlid female body size	-0.02	0.01	-2.27	**0.0234**
Time progress of experiment : *experienced* catfish	0.004	0.01	0.46	0.6479
Time progress of experiment : *highly-experienced* catfish	0.006	0.01	0.70	0.4816
**Experienced catfish**				
(Intercept)	3.83	1.06	3.62	**0.0003**
Time progress of experiment	0.003	0.004	0.74	0.4575
Catfish experience – *highly-experienced*	0.02	0.37	0.05	0.9609
Catfish experience – *naïve*	0.03	0.64	0.04	0.9672
Cichlid female body size	-0.02	0.01	-2.27	**0.0234**
Time progress of experiment : *highly-experienced* catfish	0.002	0.01	0.33	0.7397
Time progress of experiment : *naïve* catfish	-0.004	0.01	-0.46	0.6479

Supplementary Table 5: No significant effect of time progress of the experiment on the likelihood of successfully parasitising a host female. GLMM outcomes show the within-treatment fixed effects of time on the rate of cuckoo catfish parasitism success. Female total length was added as additional factor as it may affect catfish likelihood to intrude on a specific cichlid spawning to parasitise. Significant p-values ($p < 0.05$) are in bold.

	Estimate	*Std. Error*	*z*	*P*
**Naïve catfish**				
(Intercept)	-9.47	3.18	-2.98	**0.0029**
Cichlid female body size	0.02	0.01	2.67	**0.0077**
Time progress of experiment	0.06	0.03	1.92	0.0546
**Experienced catfish**				
(Intercept)	-3.95	2.28	-1.74	0.0827
Cichlid female body size	-0.01	0.01	-2.36	**0.0182**
Time progress of experiment	0.04	0.02	1.67	0.0951
**Highly-experienced catfish**				
(Intercept)	1.82	3.12	0.58	0.5600
Cichlid female body size	-0.01	0.01	-1.19	0.2330
Time progress of experiment	-0.03	0.03	-0.85	0.3980

Supplementary Table 6: No effect of time progress of the experiment on the average number of cuckoo catfish eggs found within the clutches of host females. GLMM outcomes show the within-treatment effects of time on the number of cuckoo catfish eggs found in host female clutches. Female total length was added as additional factor as it may affect catfish likelihood to intrude on a specific cichlid spawning to parasitise. Significant p-values ($p < 0.05$) are in bold.

	Estimate	*Std. Error*	*z*	*P*
**Naïve catfish**				
(Intercept)	-7.52	2.83	-2.65	**0.0080**
Cichlid female body size	0.02	0.01	2.62	**0.0088**
Time progress of experiment	0.06	0.03	1.91	0.0565
**Experienced catfish**				
(Intercept)	-1.68	1.81	-0.93	0.3547
Cichlid female body size	-0.01	0.005	-2.18	**0.0292**
Time progress of experiment	0.03	0.02	1.43	0.1530
**Highly-experienced catfish**				
(Intercept)	3.34	2.37	1.41	0.1590
Cichlid female body size	-0.01	0.01	-1.06	0.2870
Time progress of experiment	-0.03	0.03	-1.19	0.2350

Supplementary Table 7: Effects of cuckoo catfish experience and time progress on the mean number of host eggs in a clutch. Female total length and experimental bout were added as additional factors because they may affect the number of host eggs. The upper part of the table shows the GLMM results with catfish experience level 'naïve' as model reference (set with R function 'relevel'). The lower part of the table shows the results of the same GLMM with model reference set to 'experienced' cuckoo catfish using the same R function. Significant p-values ($p < 0.05$) are in bold.

	Estimate	*Std. Error*	*z*	*P*
**Naïve catfish**				
(Intercept)	-97.07	21.83	-4.42	**< 0.0001**
Cichlid female body size	2.13	0.24	8.83	**< 0.0001**
Time progress of experiment	-0.23	0.05	-4.26	**< 0.0001**
Catfish experience – *experienced*	-15.48	5.29	-2.93	**0.0034**
Catfish experience – *highly experienced*	-16.94	5.41	-3.13	**0.0018**
Experimental phase	-9.67	5.02	-1.93	0.0543
**Experienced catfish**				
(Intercept)	-113.07	21.83	-5.18	**< 0.0001**
Cichlid female body size	2.13	0.24	8.83	**< 0.0001**
Time progress of experiment	-0.23	0.05	-4.26	**< 0.0001**
Catfish experience – *highly experienced*	-1.46	5.41	-0.27	0.7869
Catfish experience – *naive*	15.48	5.29	2.93	**0.0034**
Experimental phase	-8.67	5.02	-1.93	0.0543

Supplementary Table 8: Effects of cuckoo catfish experience on the log-likelihood of missing a cichlid spawning. Experienced catfish groups missed 0% of cichlid spawning behaviours (median, range = 0% - 19%) and highly-experienced groups missed 6% (median, range = 3% - 6%) throughout their experimental trials, while naïve catfish missed a median of 11% of spawning behaviours that occurred in their experimental tanks (range = 6% - 56%). Significant p-values ($p < 0.05$) are in bold.

	Estimate	*Std. Error*	*z*	*P*
(Intercept)	-1.07	0.85	-1.26	0.2088
Catfish experience – *experienced*	-2.46	0.63	-3.87	**0.0001**
Catfish experience – *highly-experienced*	-2.02	0.55	-3.67	**0.0002**

Supplementary Table 9: Effects of cuckoo catfish experience on their time to intrude on a cichlid spawning behaviour. Significant p-values ($p < 0.05$) are in bold.

	Estimate	*Std. Error*	*t*	*P*
(Intercept)	16.60	3.83	4.33	**<0.0001**
Catfish experience – *experienced*	-3.94	1.18	-3.35	**0.0008**
Catfish experience – *highly-experienced*	-2.82	0.74	-3.79	**0.0002**

Supplementary Table 10: Effects of cuckoo catfish experience on the number of catfish individuals intruding on cichlid spawning. The upper part of the table shows the GLMM results with catfish experience level 'naïve' as model reference (set with R function 'relevel'). The lower part of the table shows the results of the same GLMM with model reference set to 'experienced' cuckoo catfish using the same R function. Significant p-values ($p < 0.05$) are in bold.

	Estimate	*Std. Error*	*t*	*P*
**Naïve catfish**				
(Intercept)	0.61	0.09	6.87	**< 0.0001**
Catfish experience – *experienced*	0.40	0.11	3.60	**0.0003**
Catfish experience – *highly-experienced*	0.008	0.12	0.95	0.9460
**Experienced catfish**				
(Intercept)	1.01	0.08	12.96	**<0.0001**
Catfish experience – *highly-experienced*	-0.39	0.14	-2.71	**0.0067**
Catfish experience – naïve	-0.40	0.11	-3.60	**0.0003**

Supplementary Table 11: Effects of catfish experience level and time progress on cichlid aggression towards cuckoo catfish. GLMM results indicate no difference in cichlid aggression toward naïve, experienced, and highly-experienced cuckoo catfish, and no change in cichlid aggression towards cuckoo catfish with experimental time progress. Significant p-values ($p < 0.05$) are in bold.

	Estimate	*Std. Error*	*z*	*P*
(Intercept)	1.71	0.58	2.95	**0.0032**
Catfish experience – *experienced*	0.24	0.31	0.79	0.4275
Catfish experience – *highly-experienced*	0.37	0.32	1.18	0.2383
Time progress of experiment	0.002	0.007	0.35	0.7233
Experimental phase	0.17	0.26	0.66	0.5099

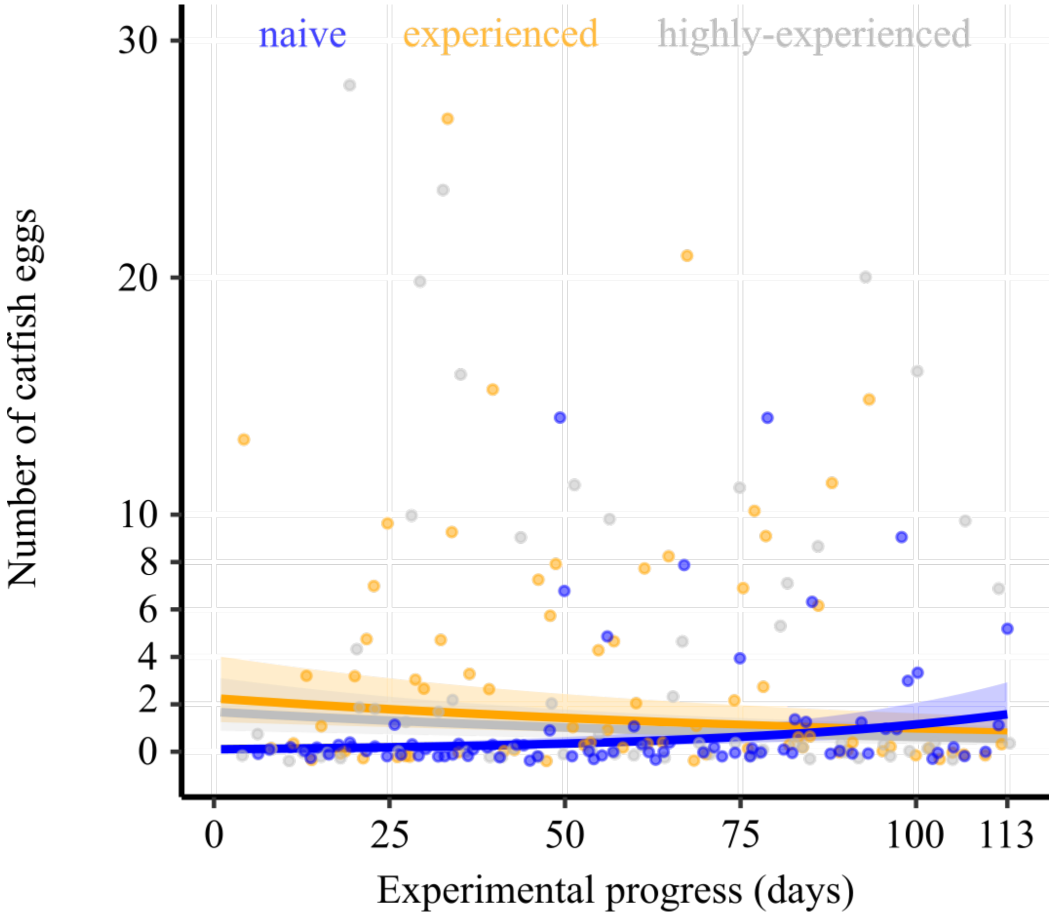

Supplementary Figure 1: Temporal dynamics of cuckoo catfish reproductive success. Coloured dots (jittered to improve visibility) show the overall number of parasite eggs in host clutches for naïve (blue), experienced (orange) and highly-experienced (grey) catfish treatments over the course of the experiment. Lines represent fitted models from the GLMM (curves for the mean estimates, and shaded areas for 1 standard error).

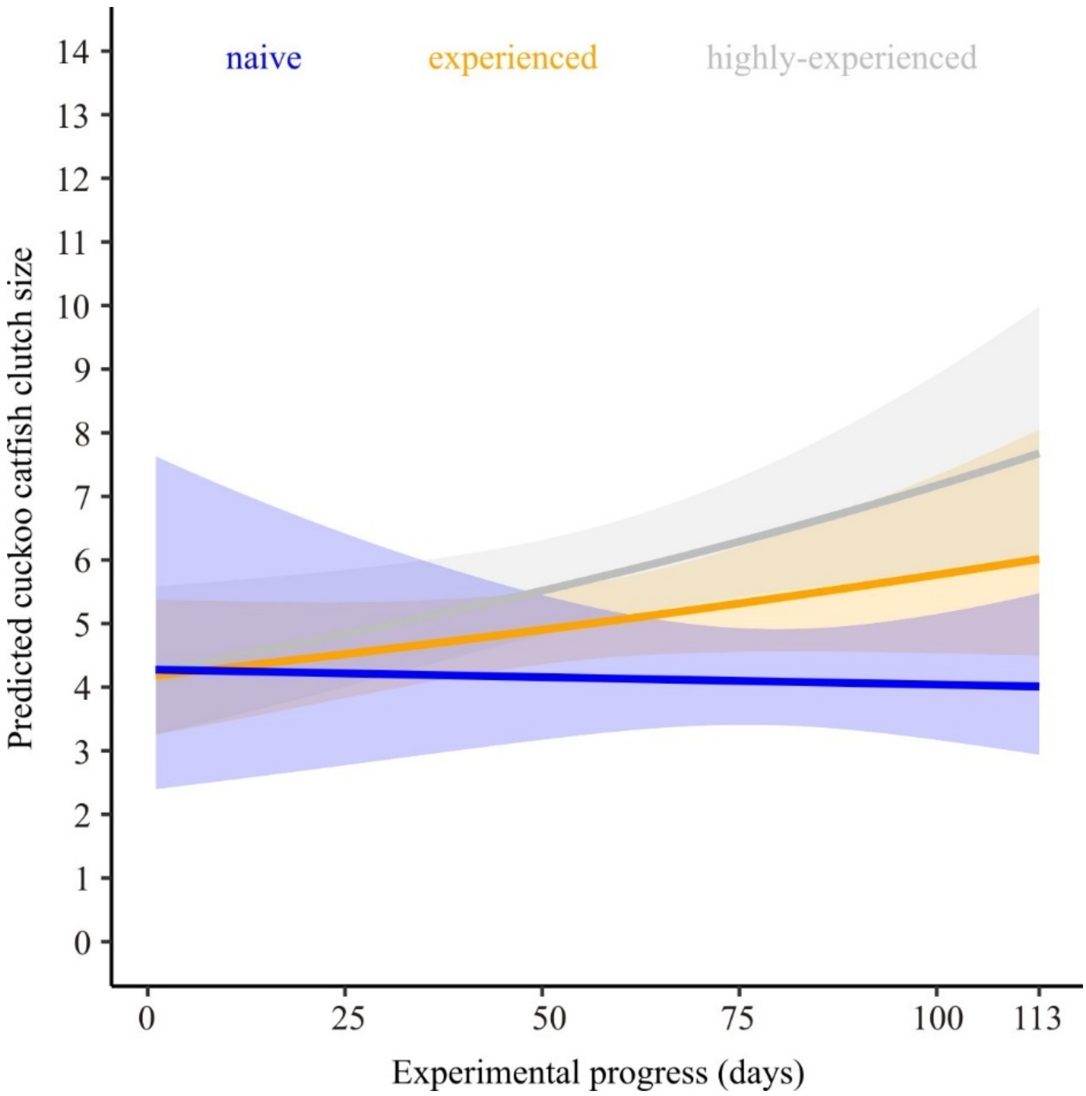

Supplementary Figure 2: Effects of catfish experience and its temporal dynamics on cuckoo catfish clutch size. Lines represent the projection of mean (± S.E.) cuckoo catfish clutch sizes for each experience level predicted by a GLMM. When successful, experienced (orange) and highly-experienced (grey) catfish managed to deposit more of their eggs among host clutches than naïve catfish (blue). Mean clutch size of catfish from the naïve treatment did not change significantly over time (GLMM, $z = -0.08$, $P = 0.937$). Catfish clutch size did not differ significantly among experience levels (GLMM, experienced: $z = -0.04$, $P = 0.967$, highly-experienced: $z = -0.01$, $P = 0.990$) and the observed differences between treatments did not change significantly over time (GLMM, interaction term 'catfish experience : time progress', experienced: $z = 0.46$, $P = 0.648$, highly-experienced: $z = 0.70$, $P = 0.482$).

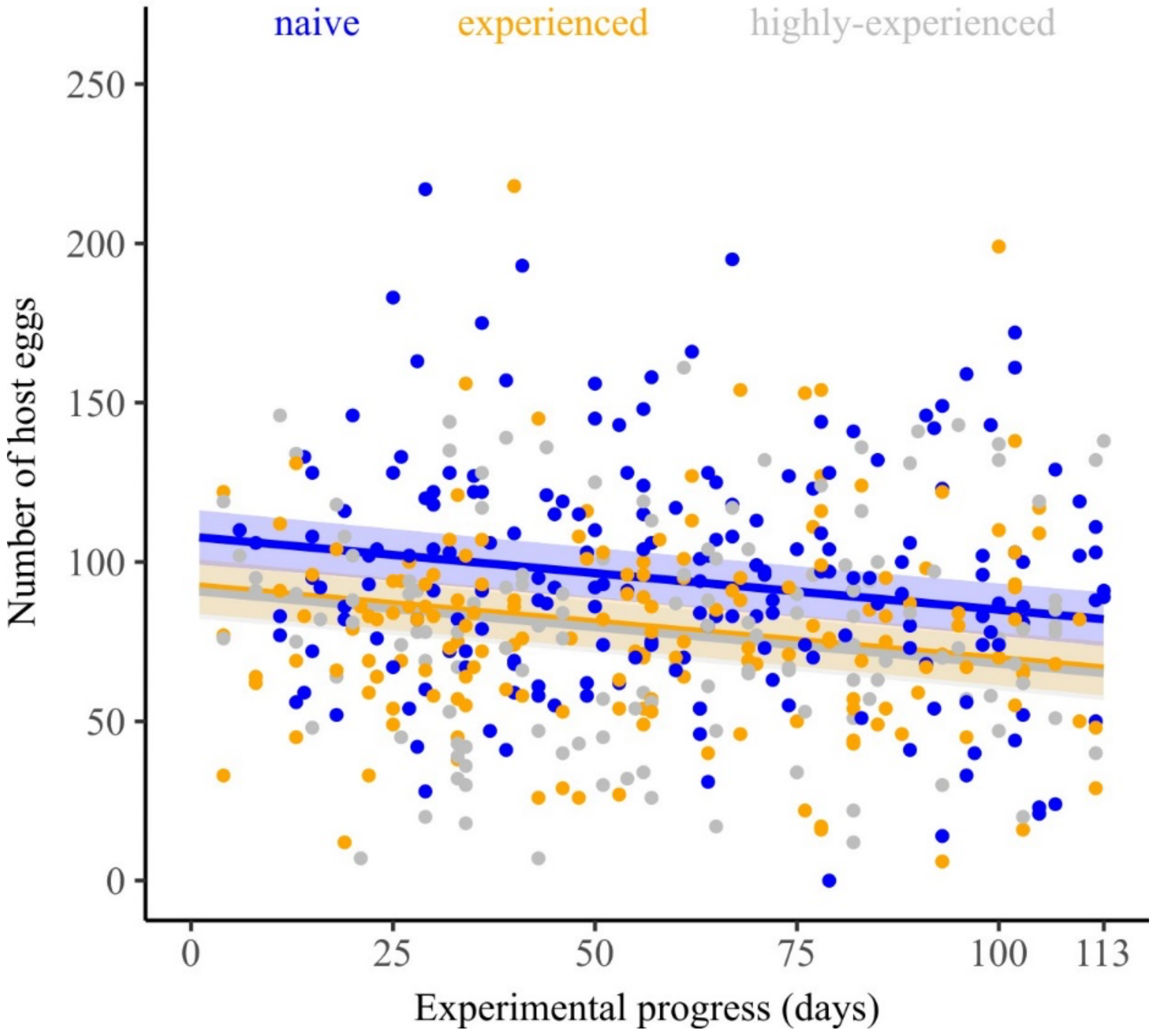

Supplementary Figure 3: Effects of catfish experience and its temporal dynamics on host clutch sizes. Temporal dynamic of the number of host eggs across cuckoo catfish experience treatments over the course of the experiment. Solid lines represent mean estimates, with 1 standard error represented by shaded areas and observed data points represented by individual dots for naïve (blue), experienced (orange) and highly-experienced (grey) cuckoo catfish treatments.
