# Supplementary material for: Individual experience as a key to success for the cuckoo catfish brood parasitism
Source: Nat Commun. 2022 Mar 31;13:1723. doi: 10.1038/s41467-022-29417-y (PMC8971504; doi:10.1038/s41467-022-29417-y)
Supplement: Supplementary file 3 — Description of Additional Supplementary Files [file 41467_2022_29417_MOESM3_ESM.pdf]

Description of Additional Supplementary Files

File Name: Supplementary Movie 1
Description: Video file with recorded behaviours.
