# Supplementary material for: Individual experience as a key to success for the cuckoo catfish brood parasitism
Source: Nat Commun. 2022 Mar 31;13:1723. doi: 10.1038/s41467-022-29417-y (PMC8971504; doi:10.1038/s41467-022-29417-y)
Supplement: Supplementary file 5 — Reporting Summary [file 41467_2022_29417_MOESM5_ESM.pdf]

nature portfolio

Corresponding author(s): Martin Reichard

Last updated by author(s): Mar 4, 2022

Reporting Summary

Nature Portfolio wishes to improve the reproducibility of the work that we publish. This form provides structure for consistency and transparency in reporting. For further information on Nature Portfolio policies, see our Editorial Policies and the Editorial Policy Checklist.

Statistics

For all statistical analyses, confirm that the following items are present in the figure legend, table legend, main text, or Methods section.

n/a	Confirmed	
☐	☒	The exact sample size (*n*) for each experimental group/condition, given as a discrete number and unit of measurement
☐	☒	A statement on whether measurements were taken from distinct samples or whether the same sample was measured repeatedly
☐	☒	The statistical test(s) used AND whether they are one- or two-sided<br>*Only common tests should be described solely by name; describe more complex techniques in the Methods section.*
☐	☒	A description of all covariates tested
☐	☒	A description of any assumptions or corrections, such as tests of normality and adjustment for multiple comparisons
☐	☒	A full description of the statistical parameters including central tendency (e.g. means) or other basic estimates (e.g. regression coefficient) AND variation (e.g. standard deviation) or associated estimates of uncertainty (e.g. confidence intervals)
☐	☒	For null hypothesis testing, the test statistic (e.g. *F*, *t*, *r*) with confidence intervals, effect sizes, degrees of freedom and *P* value noted<br>*Give P values as exact values whenever suitable.*
☒	☐	For Bayesian analysis, information on the choice of priors and Markov chain Monte Carlo settings
☐	☒	For hierarchical and complex designs, identification of the appropriate level for tests and full reporting of outcomes
☒	☐	Estimates of effect sizes (e.g. Cohen's *d*, Pearson's *r*), indicating how they were calculated

Our web collection on statistics for biologists contains articles on many of the points above.

Software and code

Policy information about availability of computer code

Data collection	No software was used.
Data analysis	All analyses were performed in the R. Package information are given in Methods and the code for all analyses and figures in available in Figshare repository (doi: 10.6084/m9.figshare.14822838.v3).

For manuscripts utilizing custom algorithms or software that are central to the research but not yet described in published literature, software must be made available to editors and reviewers. We strongly encourage code deposition in a community repository (e.g. GitHub). See the Nature Portfolio guidelines for submitting code & software for further information.

Data

Policy information about availability of data

All manuscripts must include a data availability statement. This statement should provide the following information, where applicable:

- Accession codes, unique identifiers, or web links for publicly available datasets
- A description of any restrictions on data availability
- For clinical datasets or third party data, please ensure that the statement adheres to our policy

Data and script for statistical models freely accessible on Figshare (https://doi.org/10.6084/m9.figshare.14822838.v3).

Field-specific reporting

Please select the one below that is the best fit for your research. If you are not sure, read the appropriate sections before making your selection.

☐ Life sciences ☒ Behavioural & social sciences ☐ Ecological, evolutionary & environmental sciences

For a reference copy of the document with all sections, see nature.com/documents/nr-reporting-summary-flat.pdf

Behavioural & social sciences study design

All studies must disclose on these points even when the disclosure is negative.

Study description	Experimental study in captivity with three parasite experience treatments (Synodontis multipunctatus) and hosts (Astatotilapia burtoni). Collected data on each brood and parameters of its parasitism. A subset of reproductive acts videoed and behaviour of quantitatively analysed.
Research sample	Three treatments; naïve catfish (36 individuals), age-matched experienced catfish (36 inds) and older highly-experienced catfish (36 inds), always housed with 4 male and 12 female hosts fish (Astatotilapia burtoni). 18 tanks with fish overall. Behavioural analysis based on 18 videoed spawnings. Sample size was a compromise between ethics, power and access to individuals, based on preliminary and previous studies.
Sampling strategy	Data collection over the period to collect expected number of clutches from each experimental tank. Sampling finished when the least productive treatment yielded desired number of clutches.
Data collection	The number of host and parasitic eggs was counted on a dish, written down to lab book using pencil. Three researchers collected data twice a week, typically two of them worked at the same day. They were not blind to the treatment (tanks were labelled) and were aware of working hypothesis. Host and parasite eggs are clearly distinguishable, cognitive bias at this stage was not possible.
Timing	15 Jan to 3 Aug 2020
Data exclusions	No clutch data were excluded. For behavioural data, we analysed a subset of videos that passed required quality control measures (length, visibility).
Non-participation	No fish was excluded.
Randomization	Host fish (16 per tank) were haphazardly distributed to the tanks, not as groups, but continuously seeding tanks with new individual fish. Treatment was imposed haphazardly afterwards, to retain relative balance among treatments with respect to shelf heigh, distance from the door and light (vs. randomly predetermined). Their body size was comparable.<br>For parasitic fish (6 per tank), fish were assorted to three size categories (within each treatment as it was imposed earlier by nature of the treatments) and each tank was stocked with one male and one female from each size category to minimize body size differences among tanks (given predicted impact on fecundity).

Reporting for specific materials, systems and methods

We require information from authors about some types of materials, experimental systems and methods used in many studies. Here, indicate whether each material, system or method listed is relevant to your study. If you are not sure if a list item applies to your research, read the appropriate section before selecting a response.

Materials & experimental systems

n/a	Involved in the study	
☒	☐	Antibodies
☒	☐	Eukaryotic cell lines
☒	☐	Palaeontology and archaeology
☐	☒	Animals and other organisms
☒	☐	Human research participants
☒	☐	Clinical data
☒	☐	Dual use research of concern

Methods

n/a	Involved in the study	
☒	☐	ChIP-seq
☒	☐	Flow cytometry
☒	☐	MRI-based neuroimaging

Animals and other organisms

Policy information about studies involving animals; ARRIVE guidelines recommended for reporting animal research

Laboratory animals	host cichlids (Astatotilapia burtoni), cuckoo catfish (Synodontis multipunctatus). Source of fish: Cuckoo catfish were F1 generation of commercially imported wild-caught parents (10 pairs). Host cichlids were descendant of wild fish imported from Kalambo, Zambia.

Wild animals	NA
Field-collected samples	NA
Ethics oversight	Research adhered to all national and institutional animal care and use guidelines, was administered under permit No. CZ62760203 and was approved by ethical boards of the Institute of Vertebrate Biology and the Czech Academy of Sciences (approval No. 32-2019).

Note that full information on the approval of the study protocol must also be provided in the manuscript.
